# Bitter melon juice targets molecular mechanisms underlying gemcitabine resistance in pancreatic cancer cells

**DOI:** 10.3892/ijo.2015.2885

**Published:** 2015-02-09

**Authors:** RANGANATHA R. SOMASAGARA, GAGAN DEEP, SANGEETA SHROTRIYA, MANISHA PATEL, CHAPLA AGARWAL, RAJESH AGARWAL

**Affiliations:** 1 Department of Pharmaceutical Sciences, Skaggs School of Pharmacy and Pharmaceutical Sciences, University of Colorado Anschutz Medical Campus, Aurora, CO, USA; 2 University of Colorado Cancer Center, Aurora, CO, USA

**Keywords:** pancreatic cancer, bitter melon juice, natural products

## Abstract

Pancreatic cancer (PanC) is one of the most lethal malignancies, and resistance towards gemcitabine, the front-line chemotherapy, is the main cause for dismal rate of survival in PanC patients; overcoming this resistance remains a major challenge to treat this deadly malignancy. Whereas several molecular mechanisms are known for gemcitabine resistance in PanC cells, altered metabolism and bioenergetics are not yet studied. Here, we compared metabolic and bioenergetic functions between gemcitabine-resistant (GR) and gemcitabine-sensitive (GS) PanC cells and underlying molecular mechanisms, together with efficacy of a natural agent bitter melon juice (BMJ). GR PanC cells showed distinct morphological features including spindle-shaped morphology and a decrease in E-cadherin expression. GR cells also showed higher ATP production with an increase in oxygen consumption rate (OCR) and extracellular acidification rate (ECAR). Molecular studies showed higher expression of glucose transporters (GLUT1 and 4) suggesting an increase in glucose uptake by GR cells. Importantly, GR cells showed a significant increase in Akt and ERK1/2 phosphorylation and their inhibition decreased cell viability, suggesting their role in survival and drug resistance of these cells. Recently, we reported strong efficacy of BMJ against a panel of GS cells in culture and nude mice, which we expanded here and found that BMJ was also effective in decreasing both Akt and ERK1/2 phosphorylation and viability of GR PanC cells. Overall, we have identified novel mechanisms of gemcitabine resistance in PanC cells which are targeted by BMJ. Considering the short survival in PanC patients, our findings could have high translational potential in controlling this deadly malignancy.

## Introduction

Pancreatic cancer (PanC) is a devastating disease with an extremely poor prognosis, and ranks as the fifth leading cause of cancer-related death in Western countries (1). The main reason for poor prognosis of PanC is due to high resistance to currently available chemotherapeutic agents (2). The other curative treatment of PanC is surgical resection that is possible only in 10–15% of the cases and it only slightly-improves the overall survival rate of 5% after 5 years (3). Although gemcitabine is the first-line chemotherapy for PanC patients, the response rate remains low (4). One of the major mechanisms of drug resistance in these cells is an increased energy-dependent drug efflux, resulting in decreased intracellular drug accumulation (5). Drug efflux and metabolism consume large amounts of ATP that is mainly generated via glycolysis; thereby high glycolytic rate protects cancer cells from the toxic effects of drugs by providing constant energy supply required for drug efflux and metabolism (6). Thus, the bioenergetic pathways in cancer cells could be targeted to overcome the chemoresistance and to inhibit cell proliferation and long-term survival (7). The survival signaling pathways such as PI3K/Akt and ERK that play important role in cellular functions such as proliferation, survival and metabolism, are also responsible for chemoresistance in cancer cells (8–10). Specifically, Akt activation has been directly correlated with increased rates of glucose metabolism in cancer cells (11). Akt activation stimulates anabolic metabolism, and enhances survival and suppresses apoptosis in cancer cells (12–14). Importantly, the enhanced Akt phosphorylation also confers resistance to chemotherapy (15). Duxbury *et al* have reported that Akt knockdown enhances gemcitabine chemosensitivity in PanC cells (16). All together, these studies suggest that altered metabolism and bioenergetic functions together with activated signaling pathways such as PI3K/Akt and ERK1/2 might be the major contributors to gemcitabine resistance in PanC cells, and that the agents which target them could be effective in treating gemcitabine-resistant (GR) PanC.

Bitter melon (*Momordica charantia*, Family: Cucurbitaceae) is a well-consumed vegetable in Asian countries, and is widely used for medicinal purposes; specifically, it has the ability to enhance insulin sensitivity in the body (17). There is a growing interest in bitter melon because of its beneficial effects against several diseases such as diabetes, obesity and hyperlipidemia. In addition, several studies have demonstrated that the leaf or fruit extract of bitter melon exerts antineoplastic effects against various cancers (18–21). The methanolic extract of bitter melon inhibited the colon cancer stem cell proliferation by altering energy homeostasis and inducing autophagy (22,23). Several cucurbitane-type triterpene glycosides from bitter melon have also shown strong antiproliferative activity against human breast adenocarcinoma MCF-7 cells, human colon adenocarcinoma WiDr cells, human laryngeal carcinoma HEp-2 cells, and human medulloblastoma Daoy cells (24). Importantly, bitter melon leaf extract is shown to inhibit P-glycoprotein-mediated drug efflux and to increase the efficacy of chemotherapeutic drugs in multidrug-resistant human cervical KBV1 carcinoma cells (25). Recently, we reported that bitter melon juice (BMJ) inhibits the growth of human pancreatic carcinoma cells both *in vitro* and *in vivo* through activating cellular metabolic energy sensor AMPK (26). However, BMJ efficacy against GR PanC cells has not yet been studied. Accordingly, in the present study, we investigated the mechanisms (metabolic, bioenergetic and signaling) underlying gemcitabine resistance in PanC cells, and BMJ efficacy and associated mechanism in these cells.

## Materials and methods

### Chemicals and reagents

Primary antibodies for phosphorylated and total PI3K, Akt, ERK1/2, and PTEN as well as hexokinase I and II, hypoxia inducible factor (HIF)-1α, and E-cadherin; and anti-rabbit peroxidase-conjugated secondary antibody were purchased from Cell Signaling Technology, Inc. (Beverly, MA, USA). Anti-LC3B and anti-Atg5 were from Novus Biologicals LLC (Littleton, CO, USA); anti-Beclin 1 was from BD Biosciences (San Jose, CA, USA). Anti-GLUT1 and 4 were from Abcam (Cambridge, MA, USA). β-actin antibody, gemcitabine, oligomycin, antimycin A, 2-deoxyglucose (2-DG) and carbonyl cyanide-4-(trifluoromethoxy)phenylhydrazone (FCCP) were from Sigma-Aldrich (St. Louis, MO, USA). MK-2206 was from Selleck Chemicals (Houston, TX, USA); PD98059 from EMD Millipore (Billerica, MA, USA), and LY-294002 from Adipogen Corp. (San Diego, CA, USA). ECL detection system and anti-mouse HRP-conjugated secondary antibody were from GE Healthcare (Buckinghamshire, UK). BMJ was prepared and stored as detailed recently (26). As needed, 1–4% (v/v in medium) of pure BMJ was used for cell culture studies.

### Cell culture and generation of GR PanC cells

Human pancreatic adenocarcinoma AsPC-1 and MiaPaCa-2 cells were obtained from ATCC (Manassas, VA, USA). AsPC-1 cells were cultured in Dulbecco’s Modified Eagle’s Medium (DMEM) with 10% FBS with essential amino acids; and MiaPaCa-2 cells were cultured in DMEM with 10% FBS and 2.5% horse serum under standard culture conditions (37°C, 95% humidified air and 5% CO_2_). To generate GR cell lines, at first, AsPC-1 cells were exposed to 0.1 μM concentration of gemcitabine for 3–4 days, the dead cells were removed by washing with media, and the viable cells were further exposed with 2-fold concentration of gemcitabine. The same gemcitabine treatment cycle was repeated for 3 months with increasing concentration of gemcitabine in every cycle up to 200 μM. GR MiaPaCa-2 cells were also generated by exposing to 0.1 μM gemcitabine at first and gradually increasing it up to 5 μM. Dead cells were removed regularly following each gemcitabine exposer. Both GR AsPC-1 and MiaPaCa-2 cells were grown under 5 μM gemcitabine for all the experiments.

### Cell viability assays

GR AsPC-1 cells (3×10^4^ cells/well) were seeded in complete media in 6-well plates with 5 μM gemcitabine. Next day, cells were treated with different doses of Akt and/or MEK inhibitor or BMJ for 24, 48 and 72 h. Thereafter, total cells were collected by brief trypsinization and counted using a haemocytometer. Trypan blue dye was used for assessing the number of dead cells. For apoptosis analyses, cells were stained with Annexin V/propidium iodide (PI) using Apoptosis Assay kit 2 (Molecular probes, Eugene, OR, USA) following the manufacturer’s instructions. The extent of apoptosis was determined by flow cytometry analysis of Annexin V/PI-stained cells using the fluorescence-activated cell sorting (FACS) core facility of the University of Colorado Cancer Center (Aurora, CO, USA). In another experiment, GR AsPC-1 cells were treated with 1–4% BMJ 24 and 48 h without or with pre-treatment with autophagy inhibitor 3-methyladenine (3-MA) or bafilomycin A1 (BafA1) for 2 h, and cell viability was analyzed by trypan blue assay.

### Western blotting

For western blotting, following desired treatment, total cell lysates were prepared, protein concentration estimated, and samples were subjected to SDS-PAGE on 8–16% tris-glycine gels and blotted onto nitrocellulose membrane as detailed earlier (27). Membranes were probed with specific primary antibodies overnight at 4°C followed by peroxidase-conjugated appropriate secondary antibody for 1 h at room temperature, and visualized by ECL detection system from GE Healthcare. For certain proteins, membranes were also probed with appropriate secondary IRDye-tagged antibodies and visualized using Odyssey infrared imager (LI-COR Biosciences, Lincoln, NE, USA). Membranes were also stripped and re-probed again for the protein of interest or β-actin antibody to check protein loading; however, only representative β-actin blots are shown.

### Bioenergetics analysis

XF24 Extracellular Flux Analyzer from Seahorse Bioscience, Inc. (North Billerica, MA, USA) was utilized to detect oxygen consumption rate (OCR) and extracellular acidification rate (ECAR), representing oxidative phosphorylation (OXPHOS) and glycolysis, respectively, in AsPC-1 cells (both sensitive and resistant). Briefly, cells were plated in 24-well XF cell culture microplates at 3.2×10^4^ cells/well using regular growth medium and then incubated at 37°C/5% CO_2_ for 24 h. After incubation, cells were washed twice with XF24 running medium (DMEM unbuffered assay medium adjusted to pH 7.4) and run on the XF24 analyzer to obtain real-time OCR and ECAR. As indicated four injections of compounds that modulate mitochondrial respiration and glycolysis, namely oligomycin (injection A: 1 μg/ml), FCCP (injection B: 1 μM), 2-DG (injection C: 10 mM), and antimycin A (injection D: 3 μM) were injected sequentially, in each well. Inhibitors used in the study included oligomycin that blocks ATP synthase required to determine ATP turnover rates, FCCP that uncouples mitochondria and stimulates maximal respiration and glycolysis, 2-DG that inhibits hexokinase, the first enzyme in the glycolytic pathway, and antimycin A that inhibits electron transport chain and indicates non-mitochondrial respiration (28–30). Real-time OCR and ECAR were recorded during specified programmed time periods (three readings each) as the average numbers between the injections of inhibitors mentioned above. In general, baseline OCR was calculated as respiration before injection of any compounds minus OCR after antimycin injection, and respiratory reserve capacity (RRC) was calculated using FCCP minus the basal OCR. The final data calculation was performed after the readings had been normalized with protein concentration of each well. Similarly, baseline ECAR was calculated as the recorded acidification rate during the respiratory conditions explained earlier in this section. OCR and ECAR are expressed as pmol/min/μg of protein and mpH unit change/min/μg of protein, respectively.

### Statistical analysis

All statistical analyses were performed with SigmaStat software version 2.03 (Jandel Scientific, San Rafael, CA, USA). One-way ANOVA followed by Tukey’s test was used for multiple comparisons and statistically significant difference was considered at p≤0.05.

## Results

### GR cells exhibit distinct morphology

**Figure 1:**
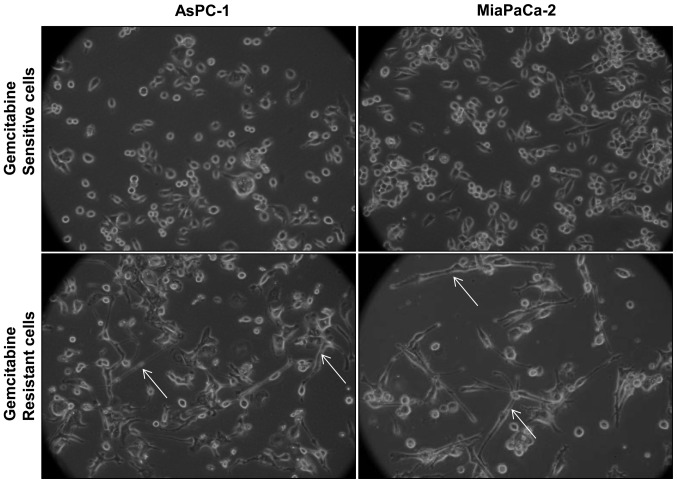
Morphology of gemcitabine-sensitive (GS) and gemcitabine-resistant (GR) cells (white arrows). GS and GR AsPC-1 and MiaPaCa-2 cells were grown to 60% confluence, and photomicrographs were captured under a light microscope (at 200x).

Morphological comparison of gemcitabine-sensitive (GS) and GR cells revealed that GR AsPC-1 and MiaPaCa-2 cells have a mixed population of small, round-shaped as well as elongated, spindle-shaped cells; however, GS counterparts mostly have small, round-shaped cells, and elongated, spindle-shaped cells were mostly absent (Fig. 1). Since both AsPC-1 and MiaPaCa-2 cells showed similar morphological features following gemcitabine exposure, for all future experiments we used AsPC-1 as a representative GR PanC cell line.

### Metabolic and molecular characterization of GR AsPC-1 cells

**Figure 2:**
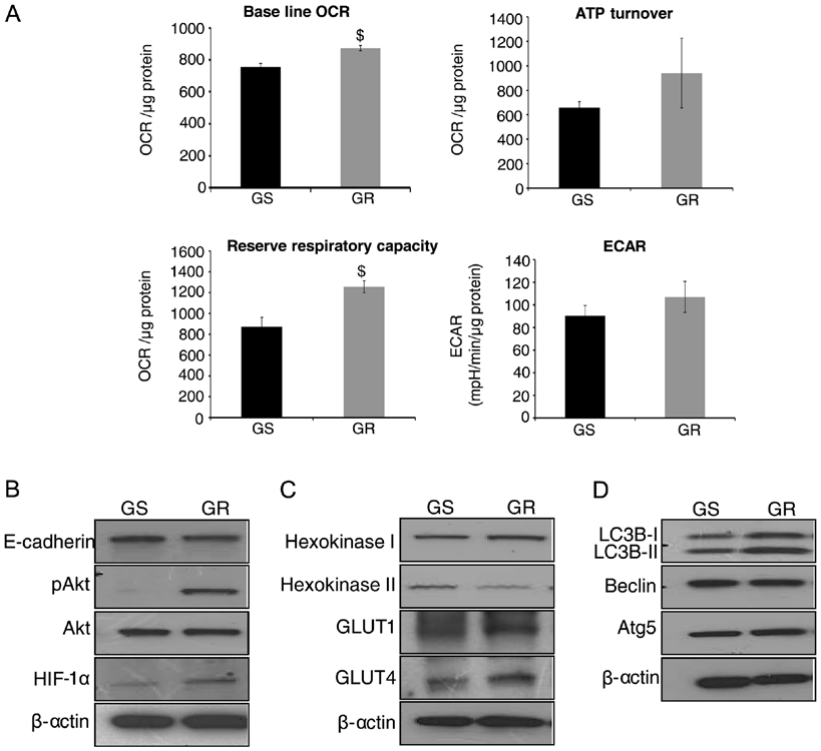
Metabolic and molecular characterization of gemcitabine-resistant (GR) pancreatic cancer (PanC) cells. (A) Gemcitabine-sensitive (GS) and GR AsPC-1 cells were plated in XF24 analyzer microplates for 24 h, and baseline oxygen consumption rate (OCR), ATP turnover, reserve respiratory capacity and extracellular acidification rate (ECAR) were measured, as detailed in Materials and methods. The representative data are presented as mean ± SEM normalized with respective protein concentration. Each experiment was performed in triplicate or quadruplicate at least twice. ^$^P<0.05. (B–D) Whole cell lysates were prepared from GS and GR AsPC-1 cells, and analyzed by western blotting for E-cadherin, pAkt, total Akt, hypoxia inducible factor (HIF)-1α, hexokinase I and II, GLUT1 and 4, LC3B, Beclin and Atg5. Protein loading was confirmed by re-probing the membranes for β-actin.

To examine the metabolic differences between GS and GR AsPC-1 cells, Seahorse XF24 Extracellular Flux Analyzer was employed, and OCR (indicative of OXPHOS) and ECAR (indicative of glycolysis) were measured. As shown in Fig. 2A (upper left panel), GR AsPC-1 cells showed an increase in baseline OCR (p≤0.05) compared with GS AsPC-1 cells. To study ATP synthesis in GR cells, OCR was determined in response to oligomycin addition, and we observed a distinct increase in ATP synthesis in GR AsPC-1 cells compared to GS AsPC-1 cells (Fig. 2A, upper right panel). RRC was also significantly higher in GR AsPC-1 cells compared to GS cells (Fig. 2A, lower left panel). Regarding glycolytic rate (indicated by ECAR), there was an increase, though statistically not significant, in ECAR in GR AsPC-1 cells compared to GS AsPC-1 cells (Fig. 2A, lower right panel). Overall, bioenergetic analyses suggested that GR AsPC-1 cells have a higher metabolic rate to possibly generate more ATP to support the chemoresistant phenotype.

We next characterized GR AsPC-1 cells to understand their morphological and metabolic differences compared to GS cells. The elongated, spindle-shaped structures in GR AsPC-1 cells suggested an epithelial-mesenchymal transition (EMT) phenotype; therefore, first we compared E-cadherin expression and found it to be slightly lower in GR compared to GS AsPC-1 cells (Fig. 2B). Akt is an important regulator of both EMT and cellular metabolism, and therefore, next we analyzed Akt phosphorylation. As shown in Fig. 2B, Akt phosphorylation (at Ser-473 site) was strongly activated in GR AsPC-1 cells with no detectable level in sensitive cells; no difference in total Akt was observed between GS and GR AsPC-1 cells. Furthermore, the expression of HIF-1α, which is downstream of Akt and is known to reduce sensitivity of PanC cells towards gemcitabine (31), was also higher in GR AsPC-1 cells (Fig. 2B).

As mentioned above, higher ATP synthesis is required to afford drug efflux from cells (7). Hexokinase is a key regulator of glycolytic flux (32), hence, we also evaluated hexokinase I and II expression. As shown in Fig. 2C, we observed a higher hexokinase I and lower hexokinase II expression in GR AsPC-1 cells compared to GS AsPC-1 cells, suggesting a preference for hexokinase I enzyme in GR cells. We also observed an increase in the protein levels of GLUT1 and 4 in GR AsPC-1 cells (Fig. 2C). To further characterize and evaluate the mechanism of gemcitabine resistance, we studied the autophagy markers in both GS and GR AsPC-1 cells. Our results indicated that LC3B-I and II protein levels were increased in GR compared to GS AsPC-1 cells without any change in the protein levels of Beclin and Atg5 (Fig. 2D).

### Akt inhibitor decreases growth and induces death in GR AsPC-1 cells

**Figure 3:**
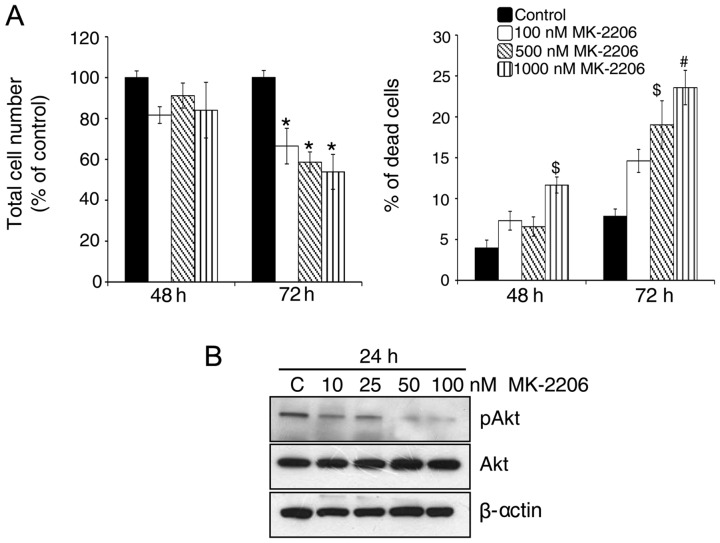
Effect of Akt inhibitor MK-2206 on cell viability in gemcitabine-resistant (GR) AsPC-1 cells. (A) GR AsPC-1 cells were treated with Akt inhibitor MK-2206 (100–1,000 nM) for 24, 48, and 72 h. At the end of each time point, both adherent and non-adherent cells were collected and processed for the determination of total cell number and dead cell percentage, as mentioned in Materials and methods. Each bar represents the mean ± SEM of three samples. ^*^P≤0.001, ^#^p≤0.01 and ^$^p≤0.05. (B) GR AsPC-1 cells were treated with MK-2206, whole cell lysates were prepared, and analyzed for phosphorylated and total Akt. Protein loading was confirmed by re-probing the membrane with β-actin antibody.

Akt is a key regulator of the balance between cell survival and apoptosis (33). As mentioned above, we observed a strong increase in Akt phosphorylation in GR AsPC-1 cells which might play an important role in drug resistance in these cells. Therefore, next, we treated GR AsPC-1 cells with an Akt inhibitor, i.e., MK-2206 (100–1,000 nM). As shown in Fig. 3A, Akt inhibition led to a significant decrease in total cell number together with an increase in dead cells in GR AsPC-1 cells especially after 72 h of its treatment. These results were consistent with a dose-dependent decrease in Akt phosphorylation levels in GR AsPC-1 cells by MK-2206 treatment (Fig. 3B), suggesting a relationship between elevated Akt phosphorylation and the resistance of AsPC-1 cells to gemcitabine.

### Akt and MEK inhibitors in combination induce cell death in GR AsPC-1 cells

**Figure 4:**
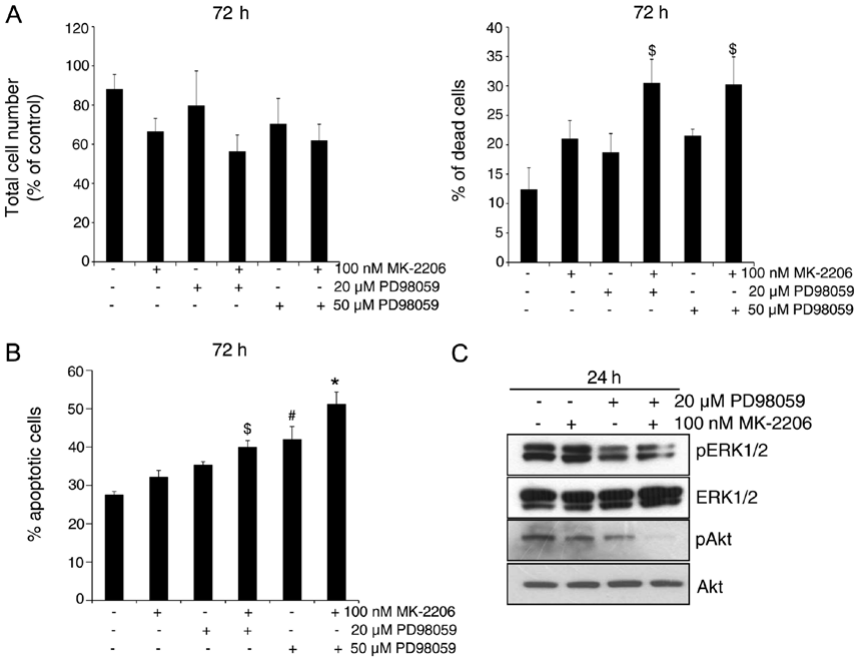
The combination of Akt inhibitor MK-2206 and MEK inhibitor PD98059 induces cell death in gemcitabine-resistant (GR) AsPC-1 cells. (A and B) GR AsPC-1 cells were treated with MK-2206 and/or PD98059 and analyzed for total cell number and percentage of dead cells by trypan blue assay, and for percentage of apoptotic cell death by Annexin V/propidium iodide (PI) staining following the procedures detailed in Materials and methods. ^*^P≤0.001, ^#^p≤0.01 and ^$^p≤0.05. (C) GR AsPC-1 cells were treated with MK-2206 and PD98059 and analyzed for phosphorylated and total ERK1/2 and Akt.

Since we did not observe a strong growth inhibitory and cell death effect of Akt inhibitor even though the Akt phosphorylation was completely inhibited by MK-2206 at 100 nM concentration (Fig. 3), we next assessed the involvement of both Akt and MEK-ERK1/2 pathways in regulating apoptosis in GR AsPC-1 cells, by employing both Akt and MEK inhibitors MK-2206 and PD98059, respectively, alone and in combination. As shown in Fig. 4A, in general, compared to each inhibitor alone, their combination resulted in a stronger cell growth inhibition and cell death in GR AsPC-1 cells. Similar observation was also evident in apoptotic cell death following MK-2206 and PD98059 treatment of GR AsPC-1 cells and a combination was better than either agent alone (Fig. 4B). Western blotting showed that indeed both ERK1/2 and Akt are strongly phosphorylated in GR AsPC-1 cells, and that treatment with MK-2206 and PD98059 reduces the phosphorylation of Akt and ERK1/2, respectively (Fig. 4C). Importantly, the combination of MK-2206 and PD98059 caused a maximum inhibition of Akt phosphorylation; however, no additional decrease in ERK1/2 phosphorylation was observed in combination compared with PD98059 alone (Fig. 4C).

### BMJ inhibits the viability of GR AsPC-1 cells via targeting PI3K/Akt pathway

**Figure 5:**
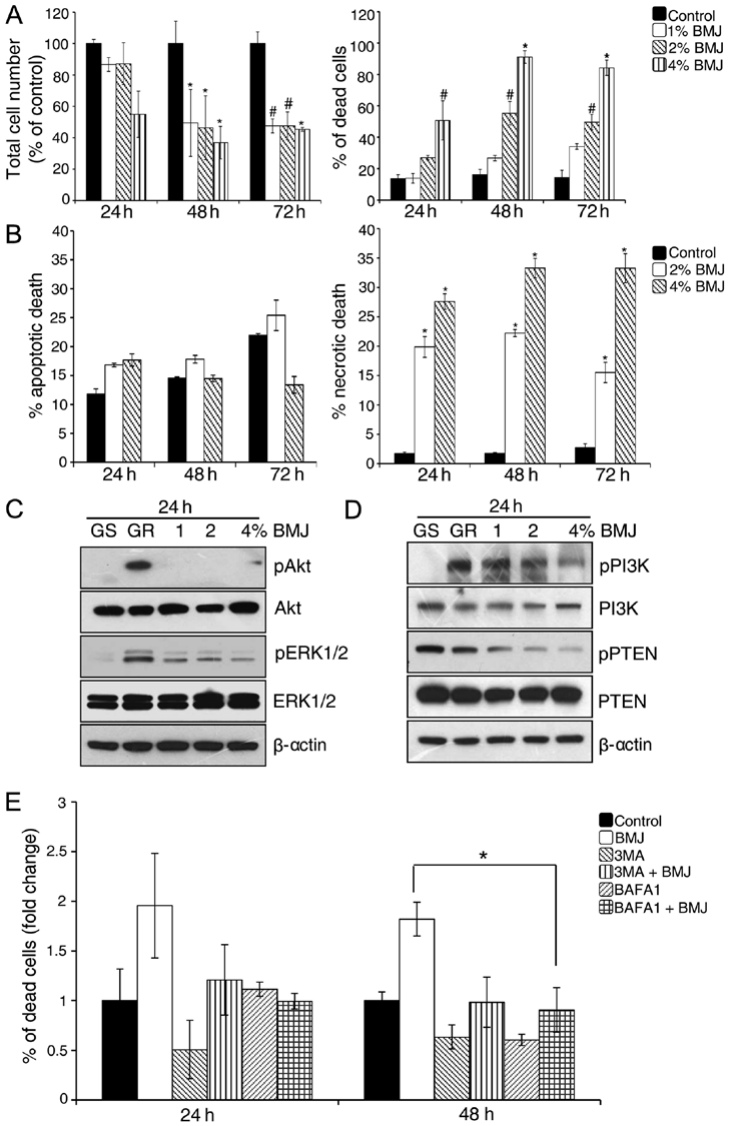
Bitter melon juice (BMJ) inhibits the viability of gemcitabine-resistant (GR) AsPC-1 cells via targeting PI3K/Akt signaling, which is autophagy-dependent. (A and B) GR AsPC-1 cells were treated with 1–4% BMJ (v/v) for 24–72 h. At the end of each time point, both adherent and non-adherent cells were collected and processed for the determination of total cell number and dead cell percentage, or percentage of apoptotic and necrotic cell death following procedures in Materials and methods. Each bar represents the mean ± SEM of three samples. ^*^P≤0.001, ^#^p≤0.01. (C and D) GR AsPC-1 cells were treated with 1–4% BMJ (v/v), total cell lysates were prepared and analyzed by western blotting for phosphorylated and total Akt, ERK1/2, PI3K and PTEN. Protein loading was confirmed by re-probing the membrane with β-actin antibody. Gemcitabine-sensitive (GS) AsPC-1 cells served as relevant control in this experiment. (E) GR AsPC-1 cells were pre-treated with early autophagy inhibitor 3-methyladenine (3-MA) or late autophagy inhibitor bafilomycin A1 (BAFA1) with or without BMJ (4%) treatment for 24 and 48 h, and dead cell percentage in each group was measured by trypan blue assay. Each bar represents the mean ± SEM of three samples. ^*^P≤0.001.

Next, we examined the effect of BMJ (1–4%) treatment on the viability of GR AsPC-1 cells. As shown in Fig. 5A, BMJ treatment significantly reduced the total cell number and increased cell death in GR AsPC-1 cells. To further characterize the BMJ-induced cell death in GR AsPC-1 cells, we stained the cells with Annexin V/PI and analyzed by FACS (Fig. 5B). Results showed that BMJ treatment did not significantly affect the apoptotic cell death but significantly increased the necrotic cell death in GR AsPC-1 cells (Fig. 5B).

PI3K/Akt signaling plays an important role in developing chemoresistance in a variety of cancer cell lines (34). We also observed an increase in Akt phosphorylation in GR AsPC-1 cells (Fig. 2B). Since we observed a significant growth inhibition by BMJ in AsPC-1 cells, we next examined the BMJ effect on Akt and related signaling molecules. Western blotting results illustrated that GR AsPC-1 cells have higher Akt, ERK1/2 and PI3K phosphorylation and a lower phosphorylated PTEN compared with GS AsPC-1 cells, and that BMJ treatment strongly reduces the Akt, ERK1/2, PI3K and PTEN phosphorylation in a dose-dependent manner without significantly affecting the total level of these molecules (Fig. 5C and D).

### BMJ induces cell death by autophagy mechanism in GR AsPC-1 cells

Since we did not observe apoptosis following BMJ treatment, we next sought to determine the role of autophagy in BMJ-induced cell death in GR AsPC-1 cells. For this purpose, we used two autophagy inhibitors, an early autophagy inhibitor 3-MA and a late autophagy inhibitor BAFA1. Results showed that the BMJ-induced cell death was compromised in the presence of both autophagy inhibitors, with stronger effect at 48 h (Fig. 5E).

## Discussion

PanC is an aggressive disease and is usually advanced at the time of diagnosis. Median survival of PanC patients post-diagnosis is <6 months and an overall 5-year survival rate is 3–5%. In 2013, ~43,920 new cases of PanC were reported in US with ~37,390 deaths (35). These statistics show that PanC is untreatable malignancy; therefore more emphasis should be placed on PanC management and control. In most PanC cases, disease relapse occurs due to chemoresistance towards drugs like gemcitabine that is the front-line therapy for PanC. Therefore, there is a critical need to understand and target mechanisms responsible for gemcitabine resistance in PanC cells. In the present study, we investigated the possible bioenergetic and molecular mechanisms underlying the gemcitabine resistance in PanC cells. Since, it is also important to identify agents that could target the molecular pathways responsible for gemcitabine resistance in PanC cells, we, for the first time, also tested the efficacy of a natural agent BMJ to target the survival of GR PanC cells. Our results are quite encouraging as BMJ effectively inhibited the proliferation and induced death in GR AsPC-1 cells. It is important to mention here that our recent studies have shown that BMJ also strongly inhibits the growth and induces apoptotic death in several PanC cell lines (GS) in culture and nude mouse xenografts (26). Therefore, BMJ could be useful against both GS and GR PanC cells.

Drug resistant cells are known to produce more ATP in comparison to the drug-sensitive cells (7); therefore, cellular bioenergetic pathways seem logical targets to overcome drug resistance in cancer cells. Role of bioenergetic pathways in gemcitabine resistance in PanC cells is not well defined; therefore, we compared the glycolytic and OXPHOS rate in GR AsPC-1 cells. We observed higher glycolysis and OXPHOS in GR AsPC-1 cells, suggesting significantly higher metabolic rate in these cells. The observed higher level of GLUT1 and GLUT4, facilitating higher glucose uptake, as well as higher hexokinase I (rate limiting enzyme during glycolysis) expression also support increased glucose metabolism in GR cells to meet higher ATP demand. In an earlier study, we reported that BMJ acts against PanC cells via activating cellular energy sensor AMPK (26); therefore, it is possible that BMJ inhibits the proliferation of GR PanC cells via enforcing energy restriction in these cells; however, further studies are needed in future to support this assumption.

Akt is a serine threonine kinase known to exert anti-apoptotic and pro-survival effects through several downstream pathways in cancer cells (36). It has been reported earlier that the inhibition of Akt activation enhances the gemcitabine sensitivity in PanC cells (4). We observed significantly higher Akt phosphorylation in GR AsPC-1 cells, and its inhibition by MK-2206 resulted in growth inhibition and induction of cell death. Besides Akt, increased level of ERK1/2 phosphorylation is also considered responsible for chemoresistance in cancer cells (37). For example, Mirmohammadsadegh *et al* reported on ERK1/2 in inducing chemoresistance in melanoma cells (38). Our data demonstrated that ERK1/2 phosphorylation is also enhanced in GR PanC cells. Importantly, inhibition of ERK1/2 phosphorylation by MEK inhibitor PD98059 also decreased the Akt phosphorylation, and we observed additional Akt inhibition when we used both MK-2206 and PD98059, suggesting crosstalk between these two pathways and that possibly Akt is downstream of MEK/ERK pathway in GR AsPC-1 cells. Importantly, the combined inhibition of both Akt and MEK/ERK pathways induced maximal apoptosis in GR AsPC-1 cells. Also notably, BMJ treatment targeted both PI3K/Akt and ERK1/2 pathways; therefore, BMJ seems to be a broad-spectrum inhibitor simultaneously targeting several signaling pathways responsible for gemcitabine resistance in PanC cells.

Previous reports have demonstrated that autophagy also contributes resistance of cancer cells towards chemotherapeutic agents by enhancing their survival and decreasing their apoptotic potential (39–41). Therefore, autophagy inhibitors have been tested in combination with chemotherapy to suppress tumor growth both *in vitro* and *in vivo* (42). We observed an increase in LC3B-I and II in GR AsPC-1 cells suggesting that autophagy could be involved in drug resistance in these cells. However, BMJ seems to induce cell death in GR AsPC-1 cells via enhancing the autophagy, as autophagy inhibitors compromised BMJ-induced cell death. Collectively, these observations suggest that further studies are needed to clearly understand the role of autophagy in conferring resistance towards gemcitabine in PanC cells as well as to understand the molecular mechanisms through which BMJ induces autophagy in these cells, as the observed effect of BMJ could also be linked to AMPK activation (26) and the resultant mTOR inhibition in PanC cells.

In summary, GR AsPC-1 cells showed an increased level of OCR and ECAR corresponding to higher ATP production in these cells. The higher expression of glycolytic proteins further confirms the increase in glucose metabolism in GR cells. Our results also revealed the important role of Akt and ERK1/2 in regulating the survival and proliferation of GR PanC cells. Present study also demonstrated the efficacy of a natural agent BMJ against GR PanC cells by targeting multiple signaling pathways including PI3K/Akt and ERK1/2. Hence, BMJ that is widely consumed as a vegetable and for health benefits could have significant efficacy against GR PanC cells. Overall, the present study reveals novel mechanisms of gemcitabine resistance in PanC cells which are targeted by BMJ; considering the poor survival rate in PanC patient, our findings could have high translational potential in controlling this deadly malignancy.
